# Gender disparities in scientific production: A nationwide assessment among physicians in Peru

**DOI:** 10.1371/journal.pone.0224629

**Published:** 2019-11-05

**Authors:** Elard Amaya, Benoît Mougenot, Percy Herrera-Añazco

**Affiliations:** 1 Universidad San Ignacio de Loyola, Vicerrectorado de Investigación, Centro de Excelencia en Investigaciones Económicas y Sociales en Salud, Lima, Perú; 2 Universidad San Ignacio de Loyola, Facultad de Ciencias Empresariales, Lima, Perú; 3 Universidad San Ignacio de Loyola, Vicerrectorado de Investigación, Unidad de Investigación para la Generación y Síntesis de Evidencias en Salud, Lima, Perú; East Carolina University Brody School of Medicine, UNITED STATES

## Abstract

**Objective:**

To determine the presence of a gender gap in the scientific production among Peruvian physicians and analyze either gap is associated with the presence of observable factors or the presence of prejudices against female physicians.

**Methods:**

We analyzed data from the National Survey of User Satisfaction in Health 2016, a nationally representative survey that collected information about medical professionals working in health institutions in Peru. The outcome of interest was the number of publications in indexed journals. We estimated the gender gap in scientific production using the Oaxaca-Blinder (OB) decomposition method.

**Results:**

From the 2216 physicians surveyed, 252 reported published at least one article in an indexed journal. From physicians with scientific production, 37.7% were women. The analysis of OB decomposition showed a gap of 2.11 indexed publications, disfavoring female physicians (p<0.01). Likewise, the explained component was 1.36 publications, representing 64.5% of the total gap (p<0.05).

**Conclusions:**

There is a gender gap in the number of publications in indexed journals among Peruvian physicians. This gap is mainly explained by observable factors, such as the years of medical practice, being an accredited researcher and being a professor.

## Introduction

Scientific research is an essential component in the training of health professionals, since its practice generates a better understanding of clinical management and, consequently, improves the healthcare provided to patients [1].

Although the number of women pursuing careers in health has significantly increased [2], several dimensions of health scientific research show gender disparities. For instances, the underrepresentation of women as speakers in medical conferences [3], the low proportion of women being first author in cases reports and articles, even in predominantly female specialties [4,5].

Most visible indicator of gender disparities in health scientific research is the number of publications in indexed journals. Medical articles produced by women has grown significantly [6–8], however there is still a substantial gender gap [9–12], where women produce a third of medical articles worldwide [13]. Several factors can explain this gap [6–8]. Most studies highlight the presence of observable factors that disadvantage women, such as barriers in access to resources and academic opportunities, as well as cultural norms that overcharge home chores for women, especially in child care responsibilities [14–19]. On the other hand, there are also non-observable factors, associated with prejudices and sexism against women, which limits their academic development [7,20].

The gender gap in the number of articles vary according to the context [21]. In low-scientific production countries, this gap is diluted and there are even cases where publications are predominantly made by women [13]. Peru is a low-scientific production country, where biomedical areas contribute the most [22]. According to Scival, during the period 2013–2018, 13,719 articles were published, where articles in "Life Sciences and Medicine" represented 50.4% of the total. On the other hand, women enrollment in medical careers has increased in recent years, the proportion of women over 17 years enrolled in medicine went from 1.5% in 2011 to 2.6% in 2016 of the total population [23].

Despite these facts, there are no studies that have measured the gender gaps in scientific production among Peruvian physicians at national level. Moreover, although several studies have measured the gender gap in the number of articles for some medical specialties, these have been limited to quantifying the raw gap without analyzing the factors that explain it [7,14,20]. To the best of our knowledge, this is the first study that measures the gender gap in the number of publications in indexed journals for a low-scientific production country.

The goals of this study are, first, to determine the existence of a gap in scientific production by gender among Peruvian physicians; and, second, to explore whether this gap is associated with the presence of observable factors or the presence of prejudices that disadvantage female physicians.

## Materials and methods

### Study design

Cross-sectional secondary analysis that used data from the National Survey of User Satisfaction in Health 2016 (ENSUSALUD-2016), which was conducted by the National Institute of Statistics and Informatics of Peru (INEI) and the National Superintendence of Health of Peru (SUSALUD). The ENSUSALUD-2016 was a nationally representative survey aimed at collecting information on the operation of the Health Services Provider Institutions (IPRESS), through the information provided by the users and providers of said services [24].

### Processes

We used the second questionnaire of the ENSUSALUD-2016, which was directed to the personnel in medicine and nursing that worked in one of the 184 IPRESS selected. The IPRESS were selected between establishments of the Ministry of Health (MINSA), Social Security in Health (EsSalud), Army/Police and Private Institutions, which had hospitalization service (I-IV level). The questionnaire was prepared by specialized personnel of SUSALUD and was conducted in person from 13^th^ May to 9^th^ July of 2016 by trained interviewers.

The ENSUSALUD-2016 used a two-stage probabilistic sampling by conglomerates and stratified by 25 administrative regions of Peru. In the first stage followed a random sampling where the unit was the IPRESS and in the second stage followed a systematically sampling where the unit was the health professionals with a minimum of one year working in the IPRESS selected.

### Population

We include in the analysis only the medical professionals who reported having at least one publication in an indexed journal, that means registered in the ISI, SCOPUS or MEDLINE databases. Physicians also reported full information about their demographic and labor-market characteristics.

### Outcome variable: Number of publications

The outcome variable was the number of publications in an indexed journal, measured as a numerical variable. This information was self-reported. In a first instance, a question was asked with a dichotomous answer (yes / no): "Have you published an original article in a journal indexed to ISI, SCOPUS or MEDLINE?" In a second instance, for physicians who offered an affirmative answer, they asked themselves: "How many original articles have you published in a journal indexed to ISI, SCOPUS or MEDLINE?" The answer to this question took values of one or more than one.

The databases cover several areas of science literature. The ISI database covers mainly the social sciences and the humanities. The Scopus database is significantly larger in size and scope, and covers more of the international literature but excludes the Humanities. The MEDLINE database covers more of the science and the biomedical literature.

### Exposure variable: Gender of the physician

The exposure of interest was the gender of the medical professional (male / female); this information was collected by direct observation of interviewees.

### Other variables

Other variables used in the analysis are: Institution in which the medical professional worked (MINSA / EsSalud / Other), age groups (23–40 / 40–55 / 55 plus), having a medical specialty (yes / no), teaching activity in higher education (yes / no) and being accredited as a researcher in the National Registry of Researchers of Science, Technology and Innovation of Peru-REGINA (yes / no). A researcher qualified as REGINA must publish academic books, articles in indexed journals, registered patents and presentation in national/international conferences.

Years practicing the medical profession, obtained by the difference between the year in which the survey was taken (year 2016) and the year he reported having obtained the university degree, later the variable was categorized into tiles. Having time for family life, which was obtained from the question "does your workload give you enough time for your personal and family life?" And was redefined in a categorical variable, in which the answer was affirmative when the Professional responded to be (totally agree / agree) and was negative when the professional gave another answer.

### Statistical analysis

We performed the analysis using Stata 15.0 statistical software (StataCorp LP, College Station, TX, USA). We considered the complex structure of the sampling using the command svy [25], weighed by conglomerate using the variable of the identifier of the IPRESS and by stratum using the variable region, and we used the survey factor expansion.

We described the continuous variables by averages and standard deviations, applying a Wald test to compare them according to gender. For the categorical variables, we used absolute frequencies and proportions, applying a χ² Pearson test for gender comparison.

We estimated the gap in scientific production by gender using the Oaxaca-Blinder (OB) decomposition method [26,27], which is expressed in the following equation:

$$\bar{{\text{Y}}^{\text{M}}}\;-\bar{{\text{Y}}^{\text{F}}}\text{=}\left(\bar{{\text{X}}^{\text{M}}}-\bar{{\text{X}}^{\text{F}}}\right){\hat{\text{β}}}^{\text{M}}\text{+}\bar{{\text{X}}^{\text{F}}}\left({\hat{\text{β}}}^{\text{M}}-{\hat{\text{β}}}^{\text{F}}\right)$$ (1)

Where the superscripts *M* and *F* represent the group of male and female, respectively. The left side of the Eq 1 shows the average gap in scientific production by gender. The first component on the right side of the Eq 1 is the explained component, which represents the proportion of the gap in scientific output that is attributed to differences in observable characteristics among male and female physicians, for our case all the adjustment variables presented previously (for example, age group, being a REGINA researcher, among others). The second component on the right side of the Eq 1 represents the proportion of the unexplained gap, which can be attributed to omitted variables (unobserved), unconscious biases, and discrimination against female physicians.

The proportions of the explained and unexplained components of the OB decomposition vary depending on the group considered as a base category (male / female), as this defines the group subject to prejudice. In our analysis we use the correction of Fortin et al. [28], which is robust to the choice of the base category.

### Ethical aspects

Our study was a secondary analysis of public data (http://portal.susalud.gob.pe/blog/base-de-datos-2016/). The interviewers solicited a verbal consent from each participant, indicating that the survey was anonymous and voluntary. Only information from the volunteer participants was collected.

## Results

### Description of the population

From the 2216 physicians surveyed, 252 reported having at least one publication in an indexed journal (11.4%). From physicians with scientific production, 37.7% were women, 43.4% worked at MINSA, 42.3% belonged to age group 23–40 years and 38.3% were in the lower title regarding time exercising the profession. Likewise, 71.7% had a specialty, 8.6% were REGINA researchers, 36.4% were professors and 32.3% had time for family life (Table 1).

**Table 1:** Characteristics of physicians with scientific production.

Characteristics		Sample size	Percentage*	CI 95%
Sex				
	Female	44	37.68	[18.82–61.20]
	Male	208	62.32	[38.80–81.18]
Institution				
	MINSA	128	43.4	[22.21–67.32]
	EsSalud	105	31.75	[12.91–59.35]
	Other	19	24.85	[9.31–51.57]
Age groups				
	24–40	67	42.26	[21.42–66.27]
	41–55	103	36.12	[19.81–56.41]
	55 or more	82	21.62	[11.84–36.18]
Years practicing the medical profession				
	Tile 1 (lower)	54	38.31	[17.86–63.95]
	Tile 2	86	31.04	[16.26–51.07]
	Tile 3 (upper)	112	30.65	[17.08–48.67]
Specialty				
	No	44	28.33	[12.49–52.26]
	Yes	208	71.67	[47.74–87.51]
REGINA researcher				
	No	235	91.45	[83.23–95.84]
	Yes	17	8.55	[4.16–16.77]
Teaching activity				
	No	127	63.57	[44.35–79.25]
	Yes	125	36.43	[20.75–55.65]
Time for family life				
	No	134	67.71	[50.70–81.04]
	Yes	118	32.29	[18.96–49.30]

* Percentage is weighted by expansion factor.

### Differences by gender in doctors with scientific output

From physicians with scientific production, we found significant differences by gender in the number of publications, the age groups, the years practicing the medical profession, being a REGINA researcher, being a professor and having time for family life. According to scientific production, male physicians had on average more publications than their female counterparts (p<0.05). In the case of age, 59% of women and 32.1% of men were concentrated in the age group of 23 to 40 years (p<0.1). With respect to time exercising the profession, 58.6% of women and 32.3% of men were in the group with the fewest years practicing the profession (p<0.1). In the case of REGINA researchers, 0.6% of women and 13.4% of men were registered as REGINA researchers (p <0.01). In the case of teachers, 11.1% of women and 51.7% of men reported working as teachers (p <0.01). Finally, with respect to family life, 11.7% of women and 44.7% of men had time to have a family life (p<0.01) (Table 2).

**Table 2:** Characteristics of physicians with at least one publication in an indexed journal by gender.

Characteristics		Female			Male			
		Sample size	Percentage**	CI 95%	Sample size	Percentage**	CI 95%	p-value
Outcome variable								0.011
	Number of publications *	44	1.91	[1.42–2.40]	208	4.02	[2.54–5.50]	
Institution								0.497
	MINSA	25	35.42	[8.48–76.45]	103	48.23	[27.37–69.73]	
	EsSalud	15	45.09	[10.00–85.85]	90	23.68	[11.33–42.99]	
	Other	4	19.5	[3.57–61.29]	15	28.08	[12.06–52.64]	
Age groups								0.097
	24–40	13	59.04	[19.77–89.39]	54	32.11	[18.43–49.75]	
	41–55	23	36.91	[9.20–77.16]	80	35.64	[21.72–52.49]	
	55 or more	8	4.05	[1.24–12.45]	74	32.25	[20.43–46.88]	
Years practicing the medical profession								0.084
	Tile 1 (lower)	11	58.55	[19.40–89.24]	43	26.07	[14.08–43.16]	
	Tile 2	17	30.83	[6.81–73.10]	69	31.17	[18.51–47.44]	
	Tile 3 (upper)	16	10.61	[3.14–30.33]	96	42.76	[28.44–58.41]	
Specialty								0.460
	No	9	21.39	[4.56–60.77]	35	32.53	[16.49–54.07]	
	Yes	35	78.61	[39.23–95.44]	173	67.47	[45.93–83.51]	
REGINA researcher								p<0.001
	No	43	99.41	[94.93–99.93]	192	86.63	[76.36–92.86]	
	Yes	1	0.59	[0.07–5.07]	16	13.37	[7.14–23.64]	
Teaching activity								p<0.001
	No	23	88.87	[69.31–96.58]	104	48.27	[34.27–62.54]	
	Yes	21	11.13	[3.42–30.69]	104	51.73	[37.46–65.73]	
Time for family life								0.004
	No	29	88.29	[67.87–96.42]	105	55.26	[40.74–68.93]	
	Yes	15	11.71	[3.58–32.13]	103	44.74	[31.07–59.26]	

* The average publications of male and female physicians were reported and Wald test was applied.

** Percentage is weighted by expansion factor.

### Oaxaca-Blinder decomposition

Through the OB decomposition, we found a gap of 2.11 indexed publications, which disadvantages women (p <0.01). The explained component was 1.36, representing 64.5% of the gap (p <0.05). On the other hand, the unexplained component was 0.75, representing the remaining 35.5%, although it was not statistically significant (Table 3).

**Table 3:** Oaxaca-Blinder decomposition.

Measure		Coefficient	Standard errors	p-value
Average number of publications estimated (male)		4.02	(0.69)	p<0.001
Average number of publications estimated (female)		1.91	(0.26)	p<0.001
Average difference		2.11	(0.77)	0.007
			
Explained component		1.36	(0.58)	0.020
	Group 1	0.57	(0.23)	0.806
	Group 2	-0.27	(1.07)	0.803
	Group 3	1.02	(1.55)	0.512
	Group 4	0.55	(0.72)	0.447
Unexplained component		0.75	(0.76)	0.323
			
Explained proportion		64.5%		
Unexplained proportion		35.5%		
			
Sample size		252		

Group1: labor institutions and specialty; Group2: age groups and time family; Group3: years practice medical profession; group4: REGINA researcher and teaching activity.

In general, the results suggest that the gender gap in scientific production is explained by differences in observable characteristics that disadvantage women, such as the years practicing medicine, being REGINA researchers and being professors. The number of years practicing medicine is the main factor, representing around 50% of the total gap.

## Discussion

Our study shows that 1 in 10 Peruvian physicians report having publications in an indexed journal. From physicians with scientific production, 4 out of 10 were women. In addition, the OB decomposition pointed out that there is a gap of 2.11 indexed publications that disadvantages women. This gender gap can be explained mainly by observable factors, such as years practicing medicine, being accredited as researchers in the REGINA and being a professor.

The frequency of publications in our study is similar to that found in a study for physicians of the residency program [1]. However, both studies are based on self-reported information, so this indicator may be overestimated. On the other hand, the low frequency of publications is compatible with the Peruvian educational system; in which the regulatory institutions of the medical profession and university authorities do not promote research efficiently [29,30]. For this reason, it seems necessary to propose a system of incentives for health scientific research [31,32], complemented with improved in the practices of research mentorship in settings such as medical undergraduate, both could improve research practice among Peruvian physicians [33–35].

Like the global trend, we find a gap in scientific production by gender [9–12]. A study that included several branches of science points out that although the gap has been narrowing, only a third of the global publications were produced by women [13]. On the other hand, a study for high-impact medical journals found that the proportion of women who figured as first authors went from 27% in 1994 to 37% in 2017 [7]. This trend has been sustained in most medical specialties and in other areas of knowledge [6–8,13], though certain variations are reported in some medical specialties [36]. A study that evaluated the gender gap in the first authors of English medical journals indicates that this gap decreased in gynecology journals, with a smaller decrease in Gastroenterology journals, presumably because of differences in the choice of specialty associated with gender [36]. These differences in the choice of the area of knowledge are a key factor, since it is more frequent to find women authors in subjects related to nursing, language, education, social work, whereas men are predominant in military subjects, and robotic or engineering sciences [13].

The causes that explain the gap in scientific production by gender are multiple and complex. These may be related to personal characteristics, or structural issues, such as fewer possibilities for women to occupy senior researchers positions and thus having a lower probability of obtaining funds to finance research, and being less likely to lead research projects or be first authors in the articles [7,8,13]. In this sense, being accredited as REGINA researchers, which includes requirements such as having published scientific articles, having participated in research projects and having advised thesis projects, and exercising university teaching, are variables that explain the gap in scientific production by gender.

With respect to time practicing the profession of medicine, several studies indicate that the time occupied in research work is related to the frequency of the production of articles [13]. In that sense, the time they have been exercising the profession could be considered as a proxy indicator of the dedication to research work, in a context in which young researchers produce fewer articles than senior researchers.

Although domestic chores, which include caring for children, are often included as a factor that could explain this gap [9,13], studies indicate that in the research, women with children and without children have the same productivity [18]. Finally, one factor that could explain this gap is the presence of prejudice or sexism, as has been suggested by other authors [7,20]. A study evaluating the applications for a laboratory position in North American universities showed that men were perceived as more competent and eligible than their female counterparts, even when the evaluators were women [20]. Although in this analysis the presence of observable factors predominated over unobserved ones, it is clear that in the academic field women suffer a series of disadvantages, such as lower likelihood of being main teachers, deans, obtaining research funds, salaries, among others [20,37–40]. This in turn suggests that unobserved factors play an essential role when discussing gender gaps.

### Limitations and strengths

Our study has some limitations. First, and mainly, the number of publications is self-reported. Although interviewers explained that the information collected in ISI, SCOPUS and MEDLINE are indexed, responses may be affected by respondents’ recall bias, so this indicator is likely to be overestimated. Second, we have included in the analysis the factors identified in the ENSUSALUD-2016, there may be other factors that influence the gender gap in scientific production and that we have not considered. Thirdly, by using a cross-sectional survey, we are not able to establish a causal relationship between the factors and the gender gap in scientific production.

The main strengths of our study are that, first, we use a method that in addition to measuring the gender gap in scientific production, quantifies the importance of the factors that explain this gap, and, second, it is based on a nationally representative survey, so the results are valuable for policymakers when proposing policies that encourage research in medical professionals.

## Conclusions

There is a gender gap in the number of articles by Peruvian physicians that disadvantages women. This gap is mainly explained by observable factors such as having less years of practicing medicine, being a REGINA researcher and being a professor. There is an opportunity to reduce this gap by promoting the career of REGINA researcher and university teaching among women who practice medicine.
